# Using video-annotation software to identify interactions in group therapies for schizophrenia: assessing reliability and associations with outcomes

**DOI:** 10.1186/s12888-017-1217-2

**Published:** 2017-02-10

**Authors:** Stavros Orfanos, Syeda Ferhana Akther, Muhammad Abdul-Basit, Rosemarie McCabe, Stefan Priebe

**Affiliations:** 1 Unit for Social and Community Psychiatry (WHO Collaborating Centre for Mental Health Service Development), Queen Mary University of London, London, UK; 2 Mental Health Research Group, University of Exeter Medical School, Exeter, UK

**Keywords:** Schizophrenia, Negative Symptoms, Group Therapy, Interactions, Video-analysis, Video-annotation, Group processes, Outcomes

## Abstract

**Background:**

Research has shown that interactions in group therapies for people with schizophrenia are associated with a reduction in negative symptoms. However, it is unclear which specific interactions in groups are linked with these improvements. The aims of this exploratory study were to i) develop and test the reliability of using video-annotation software to measure interactions in group therapies in schizophrenia and ii) explore the relationship between interactions in group therapies for schizophrenia with clinically relevant changes in negative symptoms.

**Methods:**

Video-annotation software was used to annotate interactions from participants selected across nine video-recorded out-patient therapy groups (*N* = 81). Using the Individual Group Member Interpersonal Process Scale, interactions were coded from participants who demonstrated either a clinically significant improvement (*N* = 9) or no change (*N* = 8) in negative symptoms at the end of therapy. Interactions were measured from the first and last sessions of attendance (>25 h of therapy). Inter-rater reliability between two independent raters was measured. Binary logistic regression analysis was used to explore the association between the frequency of interactive behaviors and changes in negative symptoms, assessed using the Positive and Negative Syndrome Scale.

**Results:**

Of the 1275 statements that were annotated using ELAN, 1191 (93%) had sufficient audio and visual quality to be coded using the Individual Group Member Interpersonal Process Scale. Rater-agreement was high across all interaction categories (>95% average agreement). A higher frequency of self-initiated statements measured in the first session was associated with improvements in negative symptoms. The frequency of questions and giving advice measured in the first session of attendance was associated with improvements in negative symptoms; although this was only a trend.

**Conclusion:**

Video-annotation software can be used to reliably identify interactive behaviors in groups for schizophrenia. The results suggest that proactive communicative gestures, as assessed by the video-analysis, predict outcomes. Future research should use this novel method in larger and clinically different samples to explore which aspects of therapy facilitate such proactive communication early on in therapy.

**Electronic supplementary material:**

The online version of this article (doi:10.1186/s12888-017-1217-2) contains supplementary material, which is available to authorized users.

## Background

Negative symptoms of schizophrenia are categorized along two dimensions: a reduction in expression, including lack of speech (alogia) and reduced facial expression (blunt affect); and a deficit in experiencing motivation (asociality) and pleasure (anhedonia) [1, 2]. A meta-analysis [3] found that psychological interventions delivered in groups improve these symptoms compared to treatment as usual. It was therefore concluded that interactions in a group format are clinically advantageous in the treatment of negative symptoms of schizophrenia and should be explored further [3]. If specifically helpful or unhelpful group interactions can be identified, which are linked to changes in negative symptoms, these interactions can be encouraged, or discouraged, to improve the optimal effectiveness of group therapies.

Little research has sought to investigate interactions on a moment-to-moment basis in group therapies for schizophrenia; an approach referred to as ‘interactional analysis’ (IA) [4]. In a study by Kanas and colleagues [5], the Hill Interaction Matrix [6] was used to code group interactions from verbally spoken statements. In doing so, they identified beneficial interactions fostered within the group as expression of emotions, reality testing and advice giving. However, there are at least three methodological limitations with this study. First, measurement reliability was not tested, as ratings were limited to a single researcher. Second, no attempt was made to statistically explore the relationship between interactions and clinical outcomes. Third, ratings were made in real-time through a one-way mirror, and therefore a more fine-grained analysis of group interactions was not possible.

Beck and Lewis [4] argue that the accuracy and practicality of IA can be improved by new video technologies. Research on psychiatrist-patient communication [7, 8] highlights the benefit of using video-annotation software such as ELAN [9]. This free software can be used flexibly to annotate digitally recorded data from small, inexpensive and commercially available 2D video recording devices. Crucially, annotations in ELAN can be made with a precision of up to 50 frames per second. Hence it is feasible to assume that using ELAN, verbal interactions can be accurately annotated and rated in the context of subtle nonverbal cues associated with clinical outcomes in schizophrenia [10–13].

There were two aims of this study. First was to develop and test the reliability of combining IA and ELAN video-annotation software to measure interactions in a group therapy for schizophrenia. In doing so, we sought to combine ELAN with the Individual Group Member Interpersonal Process Scale (IGMIPS) [14–16]. The second aim of this study was to assess the link between group interactions and changes in negative symptoms. This aspect of the study was exploratory. Hence interactions were compared across participants with either a clinically relevant improvement or no change in negative symptoms, and there were no fixed predictions about which IGMIPS categories would predict outcomes.

Readily available video recordings of diagnostically homogenous group therapies for individuals with schizophrenia were used in this study. These included Body-Oriented Psychotherapy (BPT) groups and Cognitive Behavioral Therapy (CBT) groups. BPT is a manualized intervention broadly aimed at reducing negative symptoms by refocusing cognitive and emotional awareness towards the body to stimulate activity [17, 18]. CBT is also a manual intervention, broadly aimed at addressing negative symptoms through thoughts, demoralized feelings and behaviors that lead to social isolation and social apathy [19]. The high audio and visual quality of the digitally recorded data was expected to contribute to high inter-rater agreement of the ratings between two independent raters.

## Methods

This study used video recordings and pre-post symptom measurements from a pool of 81 participants who attended nine separate group therapies for schizophrenia. Data from participants attending eight of the nine groups were collected as part of the NESS Trial. The NESS trial is a multi-centred randomized controlled trial (RCT) that examined the effectiveness of BPT for negative symptoms of schizophrenia in comparison to an active control group (trial registration ISRCTN84216587). Data from participants attending one of the nine groups was collected from a CBT group for this study. The BPT groups consisted of 20 sessions over 10 weeks and the CBT was 14 sessions over 10 weeks. All sessions lasted 90 min. Further details of BPT and CBT treatment groups are outlined elsewhere [17–20].

### Sample

Participants were recruited from out-patient mental health services between 2012 and 2014. Participants were aged between 18 and 65 years, had a diagnosis of schizophrenia, scored 18 or above on the negative subscale of the Positive and Negative Syndrome Scale (PANSS) [21], were willing to participate and were able to provide written informed consent.

Interactions were assessed from participants with clinically relevant changes in negative symptoms, i.e. those who met the criteria of being an ‘improver’, and participants who met the criteria for being a ‘no-changer’. Improvers were participants who had a clinically significant reduction on the PANSS negative symptoms subscale from the baseline to end of treatment assessment phase, defined as a clinically relevant reduction of at least 20% [22]. No-changers were participants who had either no change in the PANSS negative symptoms subscale, a reduction of just one point from baseline to end of treatment assessment phase, or an improvement of just one point from baseline to end of treatment assessment phase.

Participants were excluded if they had an insufficient command of English, a physical disability that impaired participation in groups, did not attend at least one session within the first and last five sessions of the group, and did not meet the criteria of an ‘improver’ or ‘no-changer’. Participants who did not have a 20% negative symptom reduction in the Clinical Assessment Interview for Negative Symptoms (CAINS) [2], a more specific measure of negative symptoms, were also excluded from the ‘improver’ category. Participants who demonstrated a reduction of more than 10% in negative symptoms measured by the CAINS were excluded from the ‘no changer’ category.

### Outcome measures

#### Positive and negative symptom scale [21]

The PANSS is a semi-structured interview, consisting of 30-items designed to measure positive, negative and general symptoms of schizophrenia. The PANSS negative symptom subscale was used for the primary outcome *‘improver status’ -* a binary outcome, which indicated whether the participants were improvers or no-changers. The negative symptom subscale includes seven items related to a difficulty in abstract thinking, poor rapport, emotional withdrawal, passive social withdrawal, lack of speech, stereotyped thinking and blunt affect.

#### Individual group member interpersonal process scale [14]

The IGMIPS [14] provides a structured coding format from which interactive behaviors made by individual group members are rated. *‘Frequency ratings’,* indicating the presence of each interactive behavioral category, were assessed as the main primary independent variable of interest. A summary of the IGMIPS categories is outlined in Table 1. Frequency ratings for each category were measured as a binary outcome (yes or no). *‘Significance ratings’,* rated on a Likert-scale, were also made to determine the intensity of a given interactive behavior. Furthermore, *‘where ratings’* were described in accordance to the ‘locational focus’ of a statement, along with *‘who’* statements referring to whom and/or what the statement is made about.

**Table 1:**
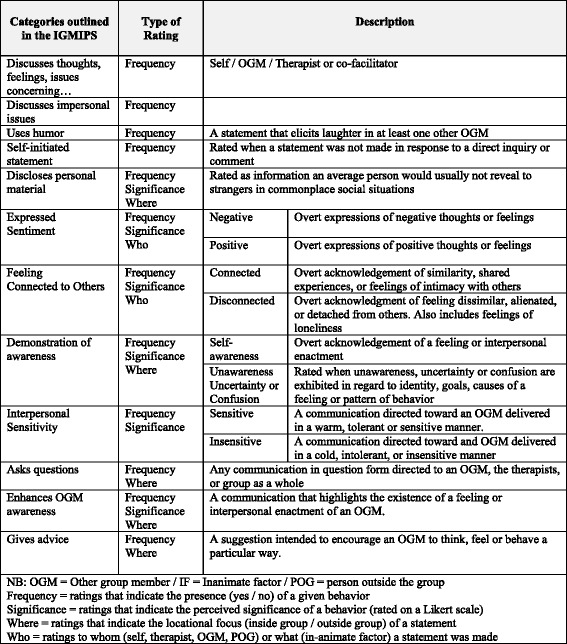
Summary of the interactive behavioral categories outlined in the Individual Group Member Interpersonal Process Scale

#### Procedure

Negative symptoms were measured before and after treatment. Baseline assessments were made within 4 weeks of the BPT and CBT groups starting and end-of-treatment assessments were made within 4 weeks of the BPT and CBT groups finishing. Six independent research assistants who had no involvement in the treatment conducted assessments. The trial through which the BPT groups were set up was a double-blind RCT. Hence, the researchers who collected this data were blind to treatment allocation, clinical outcomes and group attendance. Assessments for participants attending the CBT group were made by an unblinded researcher (SO). Hence a portion of these assessments were video-recorded and re-assessed by an independent blinded assessor. It was found that there was high inter-rater reliability (>0.82) between the two assessors before beginning the analysis of group interactions.

**Fig. 1:**
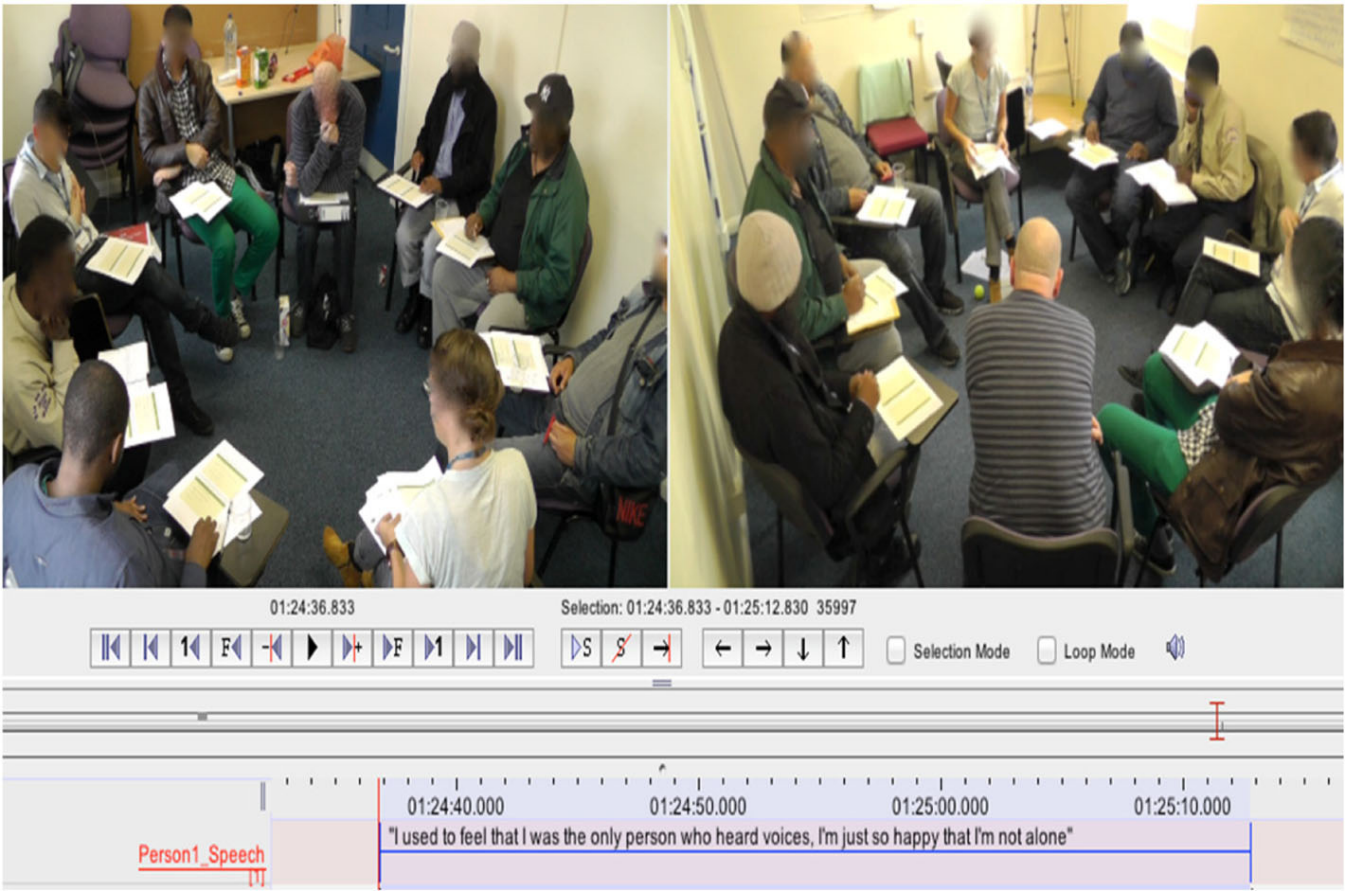
Example screenshot of video-annotation in ELAN

The analysis of group interactions occurred in two stages. In stage one, ‘individual statements’ were transcribed from participants who were identified as improvers and no-changers. These verbal statements were the primary source of material from which interactions were measured. Single ‘statements’ were defined as an utterance of three or more words, bound either by a pause of more than 10 s or by an interruption by another group member. All statements were transcribed from the video-recorded group sessions using ELAN annotation software [9]. Two video-recordings, from opposite angles in the group therapy room, were used when transcribing individual statements (see Fig. 1). Both video-recordings included their own audio file, which were used interchangeably in ELAN depending on the audio quality at each angle.

In the second stage of analysis, the group interactions were judged from the transcribed statements, using the structured coding format outlined in the IGMIPS. An adapted version of the original IGMIPS-III manual was developed for this study by the first author (SO) - see Additional file 1: Table S1 for a detailed outline of this adapted manual. Using the video-recordings of the sessions, statements were rated in the context of nonverbal cues such as tone, gaze and hand gestures. Individual statements were rated in accordance to whom the statement was being made to (i.e. self, therapist, group as a whole or other group member), the locational focus of the statement (i.e. life outside the group vs. inside the group) and whether the statement was self-initiated or elicited.

All interactions were first coded by SO. Thirty percent of statements were then rated by an independent researcher (SA), who received 3 days of training. The statements rated by SA were chosen at random and were stratified by participant. During this period a sufficient inter-rater reliability (over 80% rater agreement) was achieved.

### Statistical analysis

All analyses were conducted in STATA/SE version 12.0 (StatCorp. 2011), where *p <* 0.05 was taken to indicate statistical significance and *p <* 0.10 was taken to indicate a statistical trend. Frequency ratings, indicating the presence of interactive behaviors, were calculated as sample population percentage scores for each improver and no-changer. The sample population percentage was calculated by dividing the total number of statements rated ‘yes’ (i.e. present) for each IGMIPS category, by the total number of statements made by each participant. Scores were calculated for statements made in the first and last sessions of attendance, each of which were 90 min long.

Rater agreement between SO and SA was calculated to ensure sufficient coding reliability: this was calculated as the percentage of categories coded by both SO and SA as ‘present’. An alternative statistical test of inter-rater reliability that can account for any rater agreement that occurs due to chance, such as Cohen’s Kappa, was considered. However, the type of data measured within the IGMIPS categories varied across the different IGMIPS categories; including binary outcome data (yes/no for the ‘frequency of interactive behavior’ ratings), continuous outcome data (for the ‘significance’ ratings) and nominal outcome data (for the ‘who’ ratings). Therefore no statistical test of inter-rater reliability that could account for this variation was deemed suitable. Furthermore, the actual percentage agreement was deemed as more informative than a statistical test of inter-rater reliability, which relies on the prevalence of the measure (i.e. the number of statements rated per person).

The relationship between the frequency of interactive behavior and improver-status was explored through a series of binary logistic regression analyses. Separate analyses were conducted for each category outlined in the IGMIPS. Furthermore, separate analyses were conducted for statements coded from the first and last sessions. Significance ratings were adjusted for.

## Results

### Sample characteristics

Of the 81 participants recruited to the nine therapy groups included in this study, 17 participants met the inclusion criteria. Nine participants met the criteria for ‘improvers’ and eight participants met the criteria for ‘no-changers’. The characteristics of these participants are outlined in Table 2.

**Table 2:** Participant characteristics and number of statements made in the first and last sessions of attendance

Improver-status	% Male	Average Age	Average Change in Negative Symptoms	Statements Deleted (%)	Individual Statements Analyzed (N)		
					First session of attendance	Last session of attendance	All
Improvers	75	42.2	0.25	10	189	185	374
No-changers	89	41.7	−5.80	9.8	291	526	817

First = number of statements made in first session, Last = number of statements made in the last session, All = number of statements across both first and last sessions

In total, 1275 individual statements were coded in accordance with the categories outlined in the IGMIPS. Eighty-four of these statements (7%) were excluded from the analysis as they were rated as ‘less than 50% audible’ - i.e. less than 50% of the statement could be annotated due to inaudibility of the statement. The frequency of interactive behaviors are summarized in Table 3.

**Table 3:** Percentages of individual statements indicating the presence of IGMIPS interaction categories and corresponding significance ratings

IGMIPS Category	IMPROVERS		NO-CHANGERS	
	First session	Last session	First session	Last session
Self	64.9 (19.1)	74.0 (9.47)	78.2 (14.7)	74.2 (16.2)
Others	8.30 (6.47)	9.04 (7.55)	8.71 (9.23)	12.6 (14.9)
Therapist	26.4 (18.65)	21.7 (14.8)	14.7 (15.6)	12.6 (12.9)
Impersonal Abstract	23.2 (23.1)	17.2 (14.7)	11.6 (13.9)	13.3 (9.61)
Humor	14.6 (7.84)	18.2 (18.7)	12.3 (10.8)	21.8 (19.1)
Self-initiated	55.0 (15.6)	46.7 (9.38)	25.0 (28.70	30.2 (20.6)
Personal Information	19.1 (13.2)	26.3 (17.9)	19.5 (13.2)	28.0 (19.0)
Significance rating	2.39 (0.92)	2.01 (0.39)	2.49 (0.90)	2.17 (0.83)
Positive	32.3 (13.7)	44.7 (9.66)	32.7 (19.2)	55.4 (17.1)
Significance	2.39 (0.69)	2.43 (0.35)	2.84 (0.77)	2.47 (0.54)
Negative	16.0 (12.3)	12.5 (13.0)	14.7 (13.2)	20.4 (17.4)
Significance rating	1.69 (0.66)	1.71 (0.35)	1.62 (0.87)	1.70 (0.51)
Connected	4.54 (5.37)	8.17 (9.99)	6.47 (6.66)	10.2 (7.04)
Disconnected	0.53 (1.59)	0.96 (1.63)	0.00	3.70 (6.93)
Self-aware	16.6 (10.3)	22.9 (13.3)	14.6 (12.37)	26.4 (19.9)
Significance rating	1.39 (0.32)	1.45 (0.23)	1.23 (0.33)	1.24 (0.19)
Unaware	3.24 (5.12)	2.77 (3.27)	2.84 (4.25)	2.20 (4.25)
Significance rating	1.05 (0.10)	1.08 (0.14)	1.00 (0.00)	1.25 (0.35)
Sensitivity	5.03 (5.77)	2.32 (3.79)	1.39 (2.58)	1.81 (3.37)
Significance rating	1.30 (0.67)	1.24 (0.19)	1.25 (0.35)	1.38 (0.18)
Insensitivity	1.89 (3.19)	2.36 (3.40)	3.14 (6.55)	2.02 (5.70)
Significance rating	1.00 (0.00)	1.00 (0.00)	1.00 (0.00)	1.1 (N/A)
Asked questions	28.3 (19.0)	20.5 (14.1)	10.8 (14.4)	12.8 (17.4)
Enhanced awareness	2.35 (3.15)	5.60 (8.08)	0.00	1.34 (2.50)
Significance rating	1.5 (1.00)	1.30 (0.45)	N/A	1.00 (0.00)
Gave advice	8.94 (8.52)	6.87 (8.29)	1.22 (2.28)	3.28 (6.05)

### Inter-rater reliability

Across all IGMIPS categories, there was 96% agreement between the two independent ratings made by SO and SA. There was 92% agreement for all frequency categories rated either ‘yes’ or ‘no’. In addition, there was 98, 99 and 95% agreement for significance, locational and who items respectively. See Table 4 for a summary of the percentage agreement for each of the categories.

**Table 4:** Inter-rater percentage agreement scores for each IGMIPS category between two independent raters

IGMIPS Rating Categories	Percentage Agreement
Discusses Self	91
Discusses Others	92
Discusses Therapist	92
Discusses Abstract Issues	92
Who	83
Humor	93
Self-initiated	86
Personal Information	88
Significance	94
Where	96
Positive Sentiment	88
Who	93
Significance	96
Negative Sentiment	87
Who	99
Significance	99
Connected	93
Who	94
Disconnection	94
Who	94
Self awareness	92
Where	98
Significance	99
Self-unawareness	98
Where	100
Significance	100
Interpersonal Sensitivity	98
Significance	100
Interpersonal Insensitivity	96
Significance	100
Question	96
Where	96
Enhance OGM awareness	97
Where	100
Significance	100
Gives advice	96
Where	100

A score of ‘100’ for participant 1 in the ‘self’ category means that there was 100% agreement between the two independent raters for statements rated as ‘self’

### Linking frequency ratings of interactive behaviors and improver-status

Findings from the binary logistic regression analyses, which explored the relationship between the frequency of interactive behaviors and improver-status, are summarized in Table 5.

**Table 5:** Summary of logistic regressions exploring the relationship between the frequency of interactive behaviors and improver-status

IGMIPS Category	First session				Last session			
	Odds Ratio	SE	*P* value	95% CI	Odds Ratio	SE	*P* value	95% CI
Discusses self	0.95	0.03	0.131	0.88, 1.06	1.00	0.04	0.980	0.92, 1.10
Discusses others	0.99	0.07	0.910	0.87, 1.13	0.97	0.04	0.515	0.89, 1.06
Discusses therapist	1.04	0.03	0.181	0.98, 1.11	1.05	0.04	0.196	0.97, 1.14
Discusses impersonal/abstract	1.04	0.03	0.244	0.97, 1.10	1.03	0.04	0.519	0.94, 1.12
Uses humor	1.03	0.06	0.591	0.92, 1.15	0.99	0.03	0.678	0.94, 1.04
Self-initiated	1.06	0.03	0.046	1.00, 1.13	1.08	0.05	0.083	0.99, 1.18
Personal Information	0.98	0.05	0.643	0.88, 1.08	0.99	0.03	0.693	0.93, 1.05
Positive sentiment	0.95	0.05	0.265	0.86, 1.04	0.93	0.04	0.147	0.85, 1.02
Negative sentiment	1.01	0.04	0.862	0.93, 1.09	0.92	0.05	0.101	0.82, 1.02
Demonstrates connection	0.94	0.08	0.493	0.79, 1.12	0.97	0.06	0.624	0.86, 1.09
Demonstrates disconnection	N/A^a^				0.84	0.16	0.350	0.58, 1.22
Demonstrates self-awareness	0.97	0.06	0.625	0.87, 1.09	0.95	0.04	0.211	0.87, 1.03
Demonstrates un-awareness	1.07	0.18	0.679	0.77, 1.48	0.77	0.21	0.336	0.45, 1.32
Demonstrates sensitivity to others	1.34	0.42	0.361	0.72, 2.50	0.79	0.50	0.496	0.40, 1.57
Demonstrates insensitivity to others	0.98	0.13	0.891	0.76, 1.27	0.01	2.58	0.996	0
Asks question	1.06	0.04	0.067	1.00, 1.14	1.04	0.04	0.310	0.97, 1.11
Enhances awareness to others	N/A^a^				1.10	0.17	0.523	0.81, 1.50
Gives advice	1.42	0.27	0.067	1.00, 2.07	1.08	0.09	0.323	0.92, 1.27

^a^N/A – no statements made by participants in the no-changer category were rated as ‘disconnection’ or ‘enhanced awareness’ in the first session, therefore logistic regressions were not possible for these IGMIPS categories. *SE* standard error, *CI* confidence interval

There was a significant association between self-initiated statements and improver-status in the first session of attendance (95% CI = 1.00 to 1.13, *p <* 0.05); where a higher frequency of self-initiated statements in the first session of attendance was associated with being an ‘improver’ at the end of treatment. For statements occurring in the last session of attendance, there was a statistical trend between the frequency of self-initiated statements and improver-status (OR = 1.08, 95% CI = 0.99 to 1.18, *p =* 0.083). There was also a trend for an association between the frequency of questions (95% CI = 1.00 to 1.14, *p =* 0.067) and improver-status, and giving advice (95% CI = 1.00 to 2.07, *p =* 0.067) and improver-status; where a higher frequency of statements involving questions or giving advice in the first session of attendance was associated with being an ‘improver’ at the end of treatment.

There was no statistically significant evidence for a relationship between these variables in the last session of attendance. There was no statistically significant evidence for any of the other IGMIPS categories and improver status.

## Discussion

### Summary of findings

In line with our first hypothesis, the overall agreement between the two researchers was high. This supports the feasibility of combining a group behavioral coding scale, such as the IGMIPS, with ELAN video-annotation software. There was minimal disagreement between raters on coding the IGMIPS categories, further supporting its feasibility in measuring a range of individual group member interactions.

There was also evidence that group interactions, measured on a moment-to-moment basis using video-annotation software, were predictive of negative symptom outcomes. A higher frequency of self-initiated statements in the first session of attendance was associated with a clinically significant improvement in negative symptoms following participation in group therapy. There was also evidence for a statistical trend between more questions and advice giving in the first session of attendance, and improved odds of clinical improvements in negative symptoms.

### Strengths and limitations

This study had a number of strengths. First, stringent measures were taken to ensure that observer-rated group interactions were measured reliably, with minimal rater-bias. Participant statements were transcribed by two trained researchers to ensure minimal errors in annotating individual statements. Interactions were measured from group sessions that were recorded in high-definition with two microphone sources, for optimal visual and audio quality. Furthermore, coding reliability was assessed from a randomly selected proportion of statements coded by an independent researcher. Second, assessments of negative symptoms on 15 of the 17 participants were conducted by blinded researchers as part of a randomized controlled trial. Third, interactions were measured across multiple group psychological therapies, which have varying therapeutic orientations. Fourth, the impact of group interactions was explored in participants with clinically relevant outcomes, selected from a large pool of participants who attended multiple groups for schizophrenia.

One limitation is multiple testing. Given that separate logistic regression analyses were conducted for each of the IGMIPS categories, the chances of finding a false positive were high. A further limitation was the small sample included in the logistic regression analyses. This meant that these analyses lacked the power to detect a significant change in the odds of being an improver or no-changer following group attendance. Hence it is not possible to conclude with confidence that an effect of the frequency of interactions on improver status did not exist where there was no statistical evidence for an effect. Despite careful planning, there was also inevitably a degree of opportunism, such that data included in this study depended on the availability and quality of data that could be obtained from the NESS trial. Furthermore, the generalizability of the findings is arguably questionable, since eight of the nine groups from which data were collected were BPT groups. Given that therapist behavior wasn’t rated, it is not known how the therapeutic orientation of the intervention affected group interactions.

### Interpretation of findings

The results from this study suggest that modern video-recording devices and ELAN video-annotation software can be used to identify moment-to-moment group interactions [4]. Researchers can improve the feasibility of this approach by focusing their resources on the interactive categories identified as being most important in this study. Annotating the verbally spoken statements from the video-recorded sessions was notably the most time-consuming process. To date, clinical research has focused on advancing technologies that automatically annotate nonverbal interactive behaviors, for example 3D motion detection [23] or motion energy analysis [24]. Until recently, these approaches have only been possible to use in laboratory or restricted spaces, and therefore lack ecological validity. We therefore propose that future research would benefit more from exploring whether automatic voice recognition and transcription technologies [25] can be used with the approach described in this study. In particular, whether prosodic features of speech shown to be linked to negative symptoms of schizophrenia [26] can be used to improve the accuracy and feasibility of assessing important group interactions. Nonetheless, the research conditions, in particular the video recording equipment, did not appear to impact the clinical integrity of the treatment. Whilst this supports the feasibility of using this approach in common clinical practice, future research is needed to test the potential ease of transferability of the method described.

The results from this study support the hypothesis that interactions between group members in group therapies are linked to improvements in negative symptoms [3]. Negative symptoms are difficult to treat with conventional psychological and pharmacological medications and are linked with poor quality of life and impaired social functioning [27–29]. Hence our findings, which give insight into what type of group interactions are linked to clinically relevant improvements in this symptom domain, have important clinical implications. For example, clinicians may want to consider emphasizing activities aimed at promoting interactions related to initiating statements, asking questions or giving advice in group therapies.

The finding that self-initiation, advice giving and question asking are associated with improvements in negative symptoms is in line with research within the field of conversational analysis in schizophrenia. Within this literature, studies have found that ‘proactive’ communicative behaviors are associated with outcomes [7, 8, 23]. For example, in doctor-patient consultations, proactive gestures and asking questions have been linked with improved clinical decision making and treatment adherence [7]. Based on this literature and the results from this study, future research should explore the clinical impact of actively enhancing these types of interactions in the treatment of schizophrenia.

The method described may also be useful in identifying beneficial group interactions from the very first session of therapy. In accordance with research on group therapeutic processes [30, 31], individualized psychotherapy [32] and pharmacological treatment [33], the findings from this study highlight the importance of an initial positive response to therapy. Future research should therefore explore the impact of promoting beneficial group interactions from the very first session. In doing so, baseline participant characteristics shown to be related to clinical outcomes in schizophrenia, for example cognitive performance [34], should also be measured and accounted for.

## Conclusions

This study highlights the reliability of using video-annotation software to assess moment-to-moment interactions in a naturalistic group therapy setting for schizophrenia. Moreover, the findings suggest that behaviors assessed by this novel method are relevant for outcomes in therapies for patients with negative symptoms of schizophrenia. In particular, proactive communication identifiable from the very initial session, including self-initiated (rather than elicited) statements, advice giving and asking questions, appeared to be linked with clinically significant improvements at the end of treatment. Clinicians may therefore want to consider emphasizing activities aimed at promoting interactions related to proactive communication. Future research should explore what aspects of therapy facilitate such proactive communication early on in therapy.

## Additional file

Additional file 1: Table S1. IGMIPS Rating Adapted Manual. (DOCX 136 kb)

## Abbreviations

BPT: Body-oriented psychotherapy
CBT: Cognitive behavioral therapy
CI: Confidence interval
HIM: Hill’s interaction matrix
IF: Inanimate factor
IGMIPS: Individual group member interpersonal process scale
OGM: Other group member
PANSS: Positive and negative symptom scale
POG: Person outside the group
RCT: Randomized controlled trial

## References

[1] Fusar-Poli P, Papanastasiou E, Stahl D, Rocchetti M, Carpenter W, Shergill S, McGuire P (2015). Treatments of negative symptoms in schizophrenia: meta-analysis of 168 randomized placebo-controlled trials. Schizophr Bull.

[2] Kring AM, Gur RE, Blanchard JJ, Horan WP, Reise SP (2013). The clinical assessment interview for negative symptoms (CAINS): final development and validation. Am J Psychiatry.

[3] Orfanos S, Banks C, Priebe S (2015). Are group psychotherapeutic treatments effective for patients with schizophrenia? A systematic review and meta-analysis. Psychother Psychosom.

[4] Beck AP, Lewis, CM. The process of group psychotherapy: Systems for analyzing change. 2nd ed. Washington: American Psychological Association; 2000.

[5] Kanas N, Barr MA, Dossick S (1985). The homogeneous schizophrenic inpatient group: an evaluation using the hill interaction matrix. Small Group Behav.

[6] Hill WMF (1971). The hill interaction matrix. Personnel Guid J.

[7] McCabe R, Healey PGT, Priebe S, Lavelle M, Dodwell D, Laugharne R (2013). Shared understanding in psychiatrist–patient communication: association with treatment adherence in schizophrenia. Patient Educ Couns.

[8] Howes C, Purver M, McCabe R, Healey PGT, Lavelle M. Predicting adherence to treatment for schizophrenia from dialogue transcripts. The Association for Computational Linguistics. 2012. Paper presented at the Proceedings of the 13th Annual Meeting of the Special Interest Group on Discourse and Dialogue.

[9] Wittenburg P, Brugman H, Russel A, Klassmann A, Sloetjes H. ELAN: a professional framework for multimodality research. Proceedings of LREC. 2006

[10] Troisi A, Spalletta G, Pasini A (1998). Non-verbal behaviour deficits in schizophrenia: an ethological study of drug-free patients. Acta Psychiatr Scand.

[11] Troisi A, Pompili E, Binello L, Sterpone A (2007). Facial expressivity during the clinical interview as a predictor functional disability in schizophrenia. A pilot study. Prog Neuro-Psychopharmacol Biol Psychiatry.

[12] Brune M, Sonntag C, Abdel-Hamid M, Lehmkamper C, Juckel G, Troisi A (2008). Nonverbal behavior during standardized interviews in patients with schizophrenia spectrum disorders. J Nerv Ment Dis.

[13] Dimic S, Wildgrube C, McCabe R, Hassan I, Barnes TRE, Priebe S (2010). Non-verbal behaviour of patients with schizophrenia in medical consultations–A comparison with depressed patients and association with symptom levels. Psychopathology.

[14] Soldz S, Budman S, Davis M, Demby A (1993). Beyond the interpersonal circumplex in group psychotherapy: the structure and relationship to outcome of the individual group member interpersonal process scale. J Clin Psychol.

[15] Budman SH, Soldz S, Demby A, Davis M, Merry J (1993). What is cohesiveness? An empirical examination. Small Group Res.

[16] Davis MS, Budman SH, Soldz S. The individual group member interpersonal process scale. In: Beck AP, Lewis CM, editors. The process of group psychotherapy: systems for analyzing change. Washington: American Psychological Association; 2000. p. 283–308.

[17] Röhricht F (2015). Body psychotherapy for the treatment of severe mental disorders–an overview. Body Mov Dance Psychother.

[18] Röhricht F (2009). Body oriented psychotherapy. The state of the art in empirical research and evidence-based practice: a clinical perspective. Body Mov Dance Psychother.

[19] Staring AB, Ter Huurne MA, van der Gaag M (2013). Cognitive behavioral therapy for negative symptoms (CBT-n) in psychotic disorders: a pilot study. J Behav Ther Exp Psychiatry.

[20] Priebe S, Savill M (2016). Effectiveness of group body psychotherapy for negative symptoms of schizophrenia: multicentre randomised controlled trial. B J Psychiatry.

[21] Kay SR, Fiszbein A, Opfer LA (1987). The positive and negative syndrome scale (PANSS) for schizophrenia. Schizophr Bull.

[22] Peuskens J (1995). Risperidone in the treatment of patients with chronic schizophrenia: a multi-national, multi-centre, double-blind, parallel-group study versus haloperidol. Risperidone study group. Br J Psychiatry.

[23] Lavelle M, Healey PG, McCabe R (2013). Is nonverbal communication disrupted in interactions involving patients with schizophrenia?. Schizophr Bull.

[24] Kupper Z, Ramseyer F, Hoffmann H, Kalbermatten S, Tschacher W (2010). Video-based quantification of body movement during social interaction indicates the severity of negative symptoms in patients with schizophrenia. Schizophr Res.

[25] Bedi G, Carrillo F, Cecchi GA, Slezak DF (2015). Automated analysis of free speech predicts psychosis onset in high-risk youths. NPJ Schizophrenia.

[26] Tahir Y, Chakraborty D, Dauwels J, Thalmann N, Thalmann D, Lee J (2016). Non-verbal speech analysis of interviews with schizophrenic patients. IEEE international conference on acoustics, speech and signal processing.

[27] Blanchard JJ, Mueser KT, Bellack AS (1998). Anhedonia, positive and negative Affect, and social functioning in schizophrenia. Schizophr Bull.

[28] Hunter R, Barry S (2012). Negative symptoms and psychosocial functioning in schizophrenia: neglected but important targets for treatment. Eur Psychiatry.

[29] Velligan DI, Alphs LD (2008). Negative symptoms in schizophrenia: the importance of identification and treatment. Psychiatric Times.

[30] Burlingame GM, MacKenzie R, Strauss B, Lambert MJ (2004). Small group treatment: evidence for effectiveness and mechanisms of change. Bergin & Garfield’s handbook of psychotherapy and behavior change.

[31] Burlingame GM, Strauss B, Joyce A, Lambert MJ (2013). Change mechanisms and effectiveness of small group treatments. Bergin & Garfield’s handbook of psychotherapy and behavior change.

[32] Lambert MJ, Barley DE (2001). Research summary on the therapeutic relationship and psychotherapy outcome. Psychother Theor Res Pract Train.

[33] Priebe S, Bröker M (1997). Initial response to active drug and placebo predicts outcome of antidepressant treatment. Eur psychiatry.

[34] Farreny A (2016). Baseline predictors for success following strategy-based cognitive remediation group training in schizophrenia. J Ner Ment Dis.

