# Supplementary material for: Using video-annotation software to identify interactions in group therapies for schizophrenia: assessing reliability and associations with outcomes
Source: BMC Psychiatry. 2017 Feb 10;17:65. doi: 10.1186/s12888-017-1217-2 (PMC5301334; doi:10.1186/s12888-017-1217-2)
Supplement: Additional file 1: Table S1. — IGMIPS Rating Adapted Manual. (DOCX 136 kb) [file 12888_2017_1217_MOESM1_ESM.docx]

| IGMIPS Rating Adapted Manual | | | |
| --- | --- | --- | --- |
| Discusses whom or what? | **Self**  0 = No  1 = Yes | Rated when intention of statement is to be a communication of speaker’s thoughts/feelings/issues about oneself (including feelings about therapy, therapist or OGM).  E.G) Speaker: ‘It really bothers me that you are unable to stand up to your husband’  (NB; although focus is on OGM’s issue, it includes TP’s personal reaction to issue. Would therefore be score on ‘SELF’ and ‘OTHERS’ items).  E.G) ’I didn’t like the way people treated me here last week’ (NB; although focus of statement is the group-as-a-whole, it is the speaker’s personal reaction to the group. It does not appear that the intention was to facilitate discussion of the group issue. Hence only score as ‘SELF’).  NB: When SELF & OTHERs rated together; rate CONNECTED to others item too! | |
|  | **Others**  0 = No  1 = Yes | Rate when speaker’s communication is intended to contribute/facilitate discussion of OGM’s issue (including all OGMS, but not therapist) with the intention of a therapeutic effect;  OGM’s issue = feelings, behaviors, thoughts. Intention of therapeutic effect = mean’s that statement would have impact of sort. NB: If ‘OGM’s issue’ is vague would then would add ‘IMPER.ABS’ too; e.g. Therapist ‘its good you don’t put on weight, it’s not fair!’…‘Speaker: ‘yeah, not fair, really not fair’.  E.G: Speaker: ‘Maybe you got depressed cos my work problem reminded you of yours’ (speaker’s problem, i.e. ‘my’, mentioned, but intention of communication was clearly to help OGM understand their own issue, therefore scored on this item only).  NON-EG: Speaker ‘What do you think I should do about this issue?’ (Directed at OGM, but is not intended to facilitate a discussion about OGM’s issues).  NON-EG: Speaker ‘I don’t like what she just said’ (Reveal’s speaker’s feelings regarding OGM, but is not intended to have therapeutic effect. Therefore only scored on SELF item). | |
|  | **Therapist**  0 = No  1 = Yes | Rated when any portion of statement clearly refers to speaker’s thought’s, feelings, issues related to the therapist.  E.G: ‘I expected to like you (therapist) before we met’  E.G: ‘I will miss you (the therapist) when the group ends.  NB: Questions towards therapist also scored on this item. | |
|  | **Impersonal and Abstract Issues**  0 = No  1 = Yes | Rated when subject isn’t about self, others or therapist. Rate when speaker makes generalised/universal comments or abstract issues.  E.G: OGM: ‘I’ve come to the conclusion that people in relationships hurt each other’ (not a about self, others or therapist – rather a general ‘abstract’ statement)  E.G: Speaker: ‘I think that has to do with the fact that when you care about someone, you almost automatically expect too much’. (NB in this context, ‘you’ is used as ‘people in general’ rather than a specific group member – need to judge this!).  E.G: When general statement made about a personal experience, can be both abstract and about self – ‘After my car got booted, I realised that in general, one does not benefit from procrastination’.  E.G: general statement made in reference to OGM’s issue – also Advice giving; OGM: ‘I get anxious when I start to think about the future’…Speaker: ‘one needs to live moment by moment’ (therefore rate SELF, OTHERs….and also ADVICE) | |
| To Who is statement made? | 1 = Group  2 = Therapist  3 = Self  4 = OGM  5 = POG  6 = IF  7 = Co-fac | To whom the statement being directed towards? If statement is expressed toward more than one designee in one statement, the rater should only rate only for the designee who receives the strongest sentiment / who is most discussed | |
| Does statement elicit humor? | **Humor**  0 = No  1 = Yes | A statement that elicits laughter from any OGM/therapist…or speaker alone laughs during or after a statement.  NB: If the comment is not intended to evoke laughter, then don’t include. | |
| Speaker’s statement self-initiated? | **Self-initiated**  0 = No  1 = Yes | When speaker’s communication is made as a result of speaker’s own initiation; i.e. not a response directed toward the speaker.  Directed responses are those that specifically make clear reference to speaker…i.e. OGM ‘you are not happy today...speaker ‘yes I am’; would therefore NOT rate as self-initiated.  Indirect response are to general comments of OGM (EG. ‘it sounds like we as the group are angry’….speaker ‘that is not true’ – would rate this is as self-initiated!  Must be own initiative, not a specific response to a comment or solicitation directed by OGM/therapist.  NB; would not rate if comment is a requirement of a particular group task.  NB: statements made ‘in response’ to GM (i.e. Enhanced Awareness or giving Advice) should not be rated in this item. Not rated when self-initiated info is given in response to another unrelated ‘question’  Statement initiated in response to questions directed toward to ‘group-as-a-whole’ SHOULD be rated! E.G. Therapist – ‘how is the group today?’….Speaker – ‘I’m feeling fine.  OR Therapist – how do you think you’ll decide’…OGM – ‘I never know what to do’…Speaker – ‘I don’t either. No one does, the life is tricky’ = RATED  NON-E.G: OGM – ‘It sounds like you (speaker) get very angry at people when they tell you what to do’….Speaker – ‘well, I’m not sure if that’s true’ (Not example because statement is a direct response, even though answer is indirect). | |
| Does the speaker’s statement involve personal information? | **Personal Information**  0 = No  1 = Yes | Personal material = *information about one’s life / feelings* average person would usually not reveal to strangers in commonplace social situations – i.e. potentially *painful, potentially embarrassing, vulnerable material.* | |
|  | **Significance**  0 = non at all / 1 = little bit / 2 = somewhat / 3 = moderately / 4 = quite a bit / 5 = a great deal / 6 = extremely | | |
|  | **Where**  1 = Inside  2 = Outside | | When disclosed *material refers to speaker’s life outside the group* or in *regard to group, OGM or therapist inside the group*  E.G INSIDE designations: used when disclosed materials is in regard to speaker’s feelings toward the group, therapist, or OGM. |
| Does speaker’s statement carry a positive or negative sentiment? | **Positive Sentiment**  0 = No  1 = Yes | | Statements that express a ‘positive opinion’. Inferring whether communication of thought involves speaker feeling ‘good’ about what they are communicating; i.e when *statement is on the theme of supportive, approval, satisfaction, affection, admiration, optimism.*  Low Sig EG – “I only met him for 5min, but thought he was a nice guy” (optimistic view)  Low Sig EG – “I know I’m going to love college” (optimistic and positive sentiment)  High Sig EG – “I look forward to the group” (happy / optimistic statement)  High Sig EG – “I think your honesty is so wonderful” (explicitly a positive view |
|  | **Who**  1 = Group  2 = Therapist  3 = Self  4 = OGM  5 = POG  6 = IF  7 = Co-fac | | About whom the sentiment is being directed towards.  E.G. – Speaker: ‘I didn’t think you (OGM) were helpful last week’ = OGM designation  E.G – Speaker ‘Life sucks. Always has done’ = IF designation  E.G – Speaker ‘My sister has always been helpful to me’ = POG designation |
|  | **Significance**  *Implicit:*  1 = a little  2 = a lot  *Explicit:*  3 = slight/quite  4 = moderately  5 = strongly | | Implicit evidence = tone of voice  Explicit evidence = overtly uses adjective used to describe positive sentiment; e.g ‘I like’  3 = slight or quite (quite good, quite ok, slightly happy),  4 = moderately (good, ok, happy, agree),  5 = strongly (amazing, strongly agree) |
|  | **Negative Sentiment**  0 = No  1 = Yes | | Rate when *statement involves criticism, anger, disappointment, dissatisfaction, frustration, blame and pessimism*.  Low Sig EG – “I can’t see how this group is going to help me” (implicitly pessimistic)  Low Sig EG – “I think this discussion is fairly mindless” (negative view/opinion)  Low Sig EG – “Everyone in here is acting like a bunch of babies” (negative view/opinion)  High Sig EG – “I’m depressed” (explicitly a negative statement) |
|  | **Who**  1 = Group  2 = Therapist  3 = Self  4 = OGM  5 = POG  6 = IF  7 = Co-fac | | About whom the sentiment is being directed towards.  E.G. – Speaker: ‘I didn’t think you (OGM) were helpful last week’ = OGM designation  E.G – Speaker ‘Life suck. Always has done’ = IF designation  E.G – Speaker ‘My sister has always been helpful to me’ = POG designation |
|  | **Significance**  *Implicit:*  1 = a little  2 = a lot  *Explicit:*  3 = slight/quite  4 = moderately  5 = strongly | | Implicit evidence (tone of voice): 1 = a little, 2 = a lot  Explicit evidence (overtly uses adjective used to describe positive sentiment); 3 = slight or quite (quite bad, quite disappointed, slightly unhappy), 4 = moderately (bad, disappointed, unhappy, disagree), 5 = strongly (disagree, strongly hate) |
| Does the speaker reveal a sense of connection with others? | **Connection**  0 = No  1 = Yes | | Rated for communications that overtly reveal sense of connectedness with others; including similarity, intimacy with others, integration, bonding, agreement (agreeing with the opinion of OG) and empathy, identified shared experiences (including recall of previous behs group did together)  NB; often scored when speaker explicitly identifies shared feeling, a shared interpersonal enactment, or shared pattern of behavior.  NB; agreeing with the opinion of an OGM can be scored as connecteness  NB; whenever this is scored with an ‘inside group’ (WHERE item) designation, ‘Discusses Self’ and ‘Discusses OGM’ should be scored |
|  | **Who**  1 = Group  2 = Therapist  3 = Self  4 = OGM  5 = POG  6 = IF  7 = Co-fac | | With whom the reference of connection is made.  E.G – ‘I hate that too!’ (NB; rate SELF and OTHER)  EG – “My brother and I think exactly alike” (Is a POG designation)  EG – “I feel very close to my co-workers” (Is a POG designation)  EG – “It’s good to know people in here have the same kind of problems, and to realise that you are not the only one who feels the way you feel” (DYAD+ rating)  EG – Everyone in the group has had problems with relationships (Group) |
|  | **Disconnection**  0 = No  1 = Yes | | Rated for communications that explicitly reveal a sense of disconnectedness; feelings of alienation, dissimilarity, disagreement (including disagreeing opinions), and loneliness, identification of discrepant experiences or circumstances  NB; disagreeing with OGM regarding another group member can be scored  NB; disconnectedness expressed in regard to hypothetical situations should be scored  EG – “My boss and I don’t get on well together” (POG rating)  EG – “My wife and I have a different approach to raising our children” (POG) |
|  | **Who**  1 = Group  2 = Therapist  3 = Self  4 = OGM  5 = POG  6 = IF  7 = Co-fac | | With whom the reference of disconnection is made.  EG – “My boss and I don’t get on well together” (POG rating)  EG – “My wife and I have a different approach to raising our children” (POG)  EG – “I sometimes get lonely” (POG)  EG – “I disagree, I think the recession could have been avoided” (POG)  E-G – I never fit in with anyone in high school (POG) |
| 8) Does statement reveal a sense of self-awareness or unawareness? | **Self-awareness**  0 = No  1 = Yes | | Overt acknowledgement / stated awareness of 1) a feeling, 2) a ‘pattern’ of behavior (beh that occurs within more than one session), 3) of an interpersonal enactment (awareness to do with relationships?).  NB; carefully decide whether ‘I feel’ refers to a feeling, or is meant as ‘in my opinion…’  NB; ‘I think’ often used to describe existence of feelings; if so, rate this item!  NB; to qualify for this tem, awareness needs to be explicitly expressed – even if it appears clear from tone, non-verbal behavior, or contextual cues, it should NOT be rated unless awareness is stated!  *Outside’ designations:*  E.G ‘I like to keep things of a superficial level until I know someone well’ (demonstrates awareness of existence of pattern in her relationship)  *Inside’ designations:*  E.G. ‘I feel uncomfortable in here’ (recognition of feeling occurring within the group)  EG. ‘I don’t talk in the group until I’m settled’ (awareness of beh pattern; but no mention of co-existing/pre-existing circumstance, therefore sig rating 1)  EG ‘I am angry because you interrupted me’ (evidence of elaboration, hence at least a 2). |
|  | **Where**  1 = Inside  2 = Outside | |  |
|  | **Significance**  1 = no cause  2 = some elaboration  3 = elaboration | | In evaluating this item, immediately present, interpersonal (i.e. relationship) issues, and high levels of elaboration are most favoured. For higher significance ratings, rater should ask themselves; a) to what degree is the self-awareness elaborated? b) to what degree is the self-awareness related to interpersonal functioning? And c) where is the temporal focus (past/future, or present)?NB; patterns of behaviour can be in-session (must occur in more than one session) or outside group  1 = low intensity = awareness of feeling without suggesting cause of these occurrences  2 = medium intensity = awareness of on going pattern of behaviour or when a coexisting case of a feeling or interpersonal enactment is identified  3 = high intensity = in-depth elaboration given of identified feeling/behaviour-pattern |
|  | **Lack of awareness**  0 = No  1 = Yes | | Rated for overt unawareness, uncertainty or confusion exhibited in regard to identity, goals, or causes of a feeling or pattern of behaviour  - E.G. (of ‘outside’ designation): ‘I don’t know why I was so mean to him back them’  - E.G. (of ‘inside’ designation): ‘ I’m not sure how I feel about this group’  *Confusion examples (from confusion item)*  - E.G: OGM ‘you seem to find commitment hard’…Speaker ‘I don’t know if that’s true or not’  - E.G: Speaker ‘I think I like him, but I’m not sure yet’ |
|  | **Where** | | AS ABOVE |
|  | **Significance** | | AS ABOVE |
| Does speaker’s statement reveal a sense of Inter-personal Sensitivity or insensitivity? | **Interpersonal Sensitivity**  0 = No  1 = Yes | | Where thoughts/feelings of OGM generate a response in the speaker, resulting in a communication of understanding and caring to OGM (i.e. communication directed toward an OGM delivered in a warm, tolerant or sensitive manner).  Sensitivity is composed of = a) effect/impact of another group member b) understanding the other and c) concern/kindness  NB: a single statement may reflect both sensitivity and insensitivity; i.e. statement could be sensitive to one person and insensitive to another at the same time.  NB: Should rate the actual effect of the speaker’s response; not intended on (i.e. speaker may intend a comment to be helpful, yet if delivered in a tactless manner, the comment may embarrass the OGM).  E.G: OGM “five min ago, I was going run out of room”….Speaker “I’m glad you didn’t, you’re valued here”.  E.G. OGM “I don’t feel comfortable in terms of what I can say”…Speaker “I’m trust worthy” |
|  | **Significance** | | 1 = slight sensitive = some element of sensitiveness (i.e. reporting back what OGM said, or being kind, or mildly caring)  2 = moderately sensitive = several elements of sensitiveness  3 = very sensitive = all elements of sensitivity (awareness, understanding, kindness) |
|  | **Interpersonal Insensitivity**  0 = No  1 = Yes | | Opposite of ‘interpersonal sensitivity’; speaker is unaffected by another person’s thoughts or feelings, or if he or she is affected, there appears to be a lack of understanding and/or caring about the other. |
|  | Significance  1 = slight  2 = moderately 3 = very | | 1 = slight insensitive = some element of sensitiveness, but mainly quite cold (maybe changing convo)  2 = moderately insensitive = several elements of sensitiveness (generally reflecting on someone’s sadness/complain with frustration).  3 = very insensitive = all elements of sensitivity (awareness, understanding, kindness); complete disregard for what someone has said. |
| Is statement a question? | **Asked question**  0 = No  1 = Yes | | Any communication in question form directed to an OGM, the therapists, or group as a whole |
|  | **Where**  1 = Inside  2 = Outside | |  |
| Does the statement enhances OGM awareness? | **Enhance awareness**  0 = No  1 = Yes | | A statement in which the speaker highlights a feeling, pattern of behaviour, or interpersonal enactment of an OGM.  Need to consider 1) the degree to which the speaker elaborates on OGM-awareness , 2) if the OGM awareness is related to interpersonal functioning and 3) the temporal focus of the OGM-awareness.  E.G (outside group) “remember you told your roommate before she moved in that you were allergic to cigarette smoke’  E.G (outside group) “it seems like you go through cycles of high and low energy”  E.G (inside group) “you look sad today” |
|  | **Where**  1 = Inside  2 = Outside | |  |
|  | **Significance**  1 = low intensity  2 = medium intensity  3 = high intensity | | Significance ratings are scored higher for suggestion of causes.  1 = low intensity (awareness of OGM feeling without suggesting cause of these occurrences)  2 = medium intensity (awareness of on going pattern of behaviour in OGM or when a coexisting case of a feeling or interpersonal enactment of OGM is identified)  3 = high intensity (in-depth elaboration given of identified feeling/beh-pattern in OGM) |
| Is statement intended to give advice? | **Gives advice**  0 = No  1 = Yes | | A directive/suggestion intended to encourage an OGM to think, feel or behave a particular way. |
|  | **Where**  1 = Inside  2 = Outside | |  |
